# Supplementary material for: Comprehensive Annotation and Functional Exploration of MicroRNAs in Lettuce
Source: Front Plant Sci. 2021 Dec 24;12:781836. doi: 10.3389/fpls.2021.781836 (PMC8739914; doi:10.3389/fpls.2021.781836)
Supplement: Supplementary file 2 [file Data_Sheet_8.ZIP › GSM2230747.plot/Lsa-miRN1664_Lsat_1_v5_gn_3_84440.1_2887_TPlot.pdf]

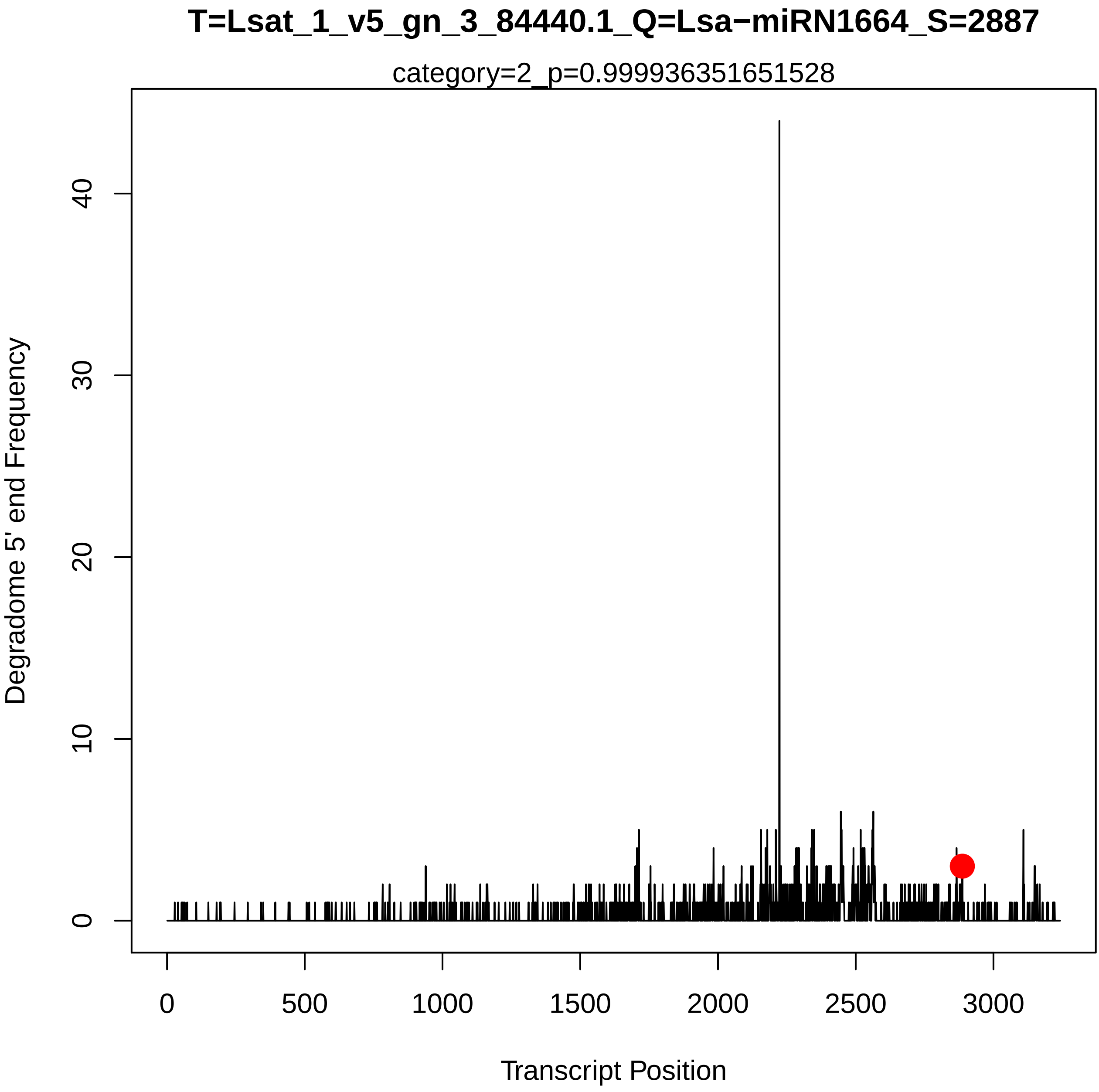
T=Lsat_1_v5_gn_3_84440.1_Q=Lsa−miRN1664_S=2887
category=2_p=0.999936351651528
Degradome 5' end Frequency
0
10
20
30
40
0
500
1000
1500
2000
2500
3000
Transcript Position
